# Revealing the Perfect Smile: How Philtrum Length Shapes Lip Beauty

**DOI:** 10.1007/s00266-025-04752-9

**Published:** 2025-03-05

**Authors:** Rui Zeng, Emily Glaue, Nicholas Moellhoff, Kyu-Ho Yi, Alexandra Anker, Philipp Unbehaun, Vanessa Brébant, Wei-Jin Hong, Lukas Prantl, Konstantin Frank

**Affiliations:** 1 Department for Hand, Plastic and Aesthetic Surgery, Ludwig – Maximilian University Munich, Munich, Germany; 2 Department of Otorhinolaryngology, University Hospital Regensburg, Regensburg, Germany; 3 Maylin Clinic (Apgujeong), Seoul, Korea; 4 Aesthetic, Hand and Reconstructive Surgery, University Center for Plastic, University Hospital Regensburg, Regensburg, Germany; 5 Department of Plastic and Reconstructive Surgery, Guangdong Second Provincial General Hospital, Guangzhou, China; 6 Department of Plastic, Hand, and Reconstructive Surgery, University Hospital Regensburg, Regensburg, Germany

**Keywords:** Lip aesthetics, Philtrum length, Ethnic beauty standards, Facial proportions

## Abstract

**Background:**

Lips significantly influence facial aesthetics, driving the growing demand for lip augmentation procedures, particularly the bullhorn lip lift. As aesthetic medicine becomes more diverse with globalization, understanding how ethnicity affects aesthetic perceptions of lips is increasingly important.

**Objectives:**

To examine the philtrum length and lip proportions in the context of different ethnic backgrounds, recognizing the increasing globalization of beauty standards.

**Methods:**

Five frontal facial images of females from diverse ethnic groups—African, Asian, Caucasian, Latino, and Middle Eastern—were generated using Midjourney and were edited to simulate various degrees of philtrum shortening and corresponding changes in the upper vermillion height and maxillary incisor show. An online survey was then conducted to gather participant feedback on the aesthetic appeal of the lips in each image, using a 5-point Likert scale.

**Results:**

A total of 570 respondents participated in the study. Overall, the most preferred lips were those with a 1 mm incisor show in African and Asian images, while lips with a 0.5 mm incisor show were favored in Caucasian and Latino images, and 0 mm in Middle Eastern images. The least favored lips were predominantly those with a 4 mm incisor show. Preferences across age and ethnic groups primarily favored the 0 mm, 0.5 mm, and 1 mm incisor shows.

**Conclusions:**

This study provides valuable insights into the complex relationship between philtrum length and the perception of beauty. The findings emphasize the importance of achieving a balanced aesthetic that aligns with the patient’s cultural background and personal preferences.

**Level of Evidence V:**

This journal requires that authors assign a level of evidence to each article. For a full description of these Evidence-Based Medicine ratings, please refer to the Table of Contents or the online Instructions to Authors www.springer.com/00266.

## Introduction

The lips are a vital anatomical feature that contributes significantly to the attractiveness of the lower third of the face. Their volume, shape, color, and movement play a crucial role in expressing emotions and, consequently, in defining an individual's overall facial beauty.[1–5] The appearance of the lips can even impact self-esteem, underscoring their importance in aesthetic medicine. Previous studies have extensively discussed the ideal features of lips, highlighting the significance of the relationship between the upper and lower lips and the chin in determining facial attractiveness.[4, 6–10]

The popularity of lip augmentations for aesthetic purposes is steadily increasing, driven by the desire to enhance facial beauty through various techniques. These range from the application of lipstick to more advanced procedures such as volumization using topical agents and hyaluronic acid-based soft tissue fillers, which not only add volume but also assist in shaping and contouring the lips. Surgical options, particularly the bullhorn lift, are also on the rise. This surgical procedure involves the excision of skin beneath the nose to reduce the length of the philtrum—a feature that can be genetically long in young patients or also elongated due to aging.[11–19] As this procedure gains popularity, it is essential to investigate the optimal philtrum length that is considered aesthetically pleasing. As the demand for lip augmentation procedures continues to grow, it is crucial to establish a more comprehensive understanding of what constitutes an aesthetically pleasing philtrum length and lip proportion. The influence of ethnicity on perceptions of beauty and attractiveness is a crucial aspect to explore, especially in relation to lip aesthetics. With globalization and increased migration, the patient population in aesthetic medicine has become increasingly diverse.[1] This diversity presents both a challenge and an opportunity for plastic surgeons, dermatologists, and aesthetic medicine professionals, who must now understand the varied needs of patients from different ethnic and cultural backgrounds. Understanding how ethnicity, along with demographic and occupational factors, influences perceptions of beauty is essential for delivering personalized and culturally sensitive treatments. Thus, the objective of the present study is to examine the philtrum length and lip proportions in the context of different ethnic backgrounds, recognizing the increasing globalization of beauty standards.

## Materials and Methods

### Philtrum Length Simulation

Midjourney (Midjourney, San Francisco, CA, USA) is an advanced artificial intelligence platform that generates high-quality, photorealistic images based on user-provided textual prompts. The platform employs deep learning models, specifically a generative adversarial network (GAN), which consists of two neural networks: a generator and a discriminator. These networks work in tandem to produce images that closely match the input description.

For this study, the prompts were crafted to emphasize “attractive lips” in accordance with aesthetic standards characteristic of five ethnic groups—African, Asian, Caucasian, Latino, and Middle Eastern. The process involves the following steps:

- **Prompt Input:** The user provides a textual description (prompt) detailing the desired attributes of the generated image, including specific features such as lip shape, size, symmetry, and skin tone.
- **Image Synthesis:** The AI model interprets the prompt and generates an initial image by creating pixel-level patterns that match the described features. This process is guided by the training data the model has been exposed to, which includes millions of diverse facial images.
- **Iterative Refinement:** The GAN refines the generated image by iterating through multiple cycles of generation and evaluation. The discriminator network evaluates the realism of the generated image compared to the input description, and the generator improves the image accordingly.
- **Ethnic Group Aesthetic Standards:** The platform was instructed to include features reflecting cultural and aesthetic norms typically associated with each ethnic group, such as variations in lip fullness, shape, and symmetry. These norms are inferred based on the training dataset's representation of diverse populations.
- **Final Output:** The final images produced by the model are photorealistic and represent the requested ethnic-specific features. These images are then reviewed by the researchers to ensure they meet the study's criteria and objectives.

**Fig. 1:**
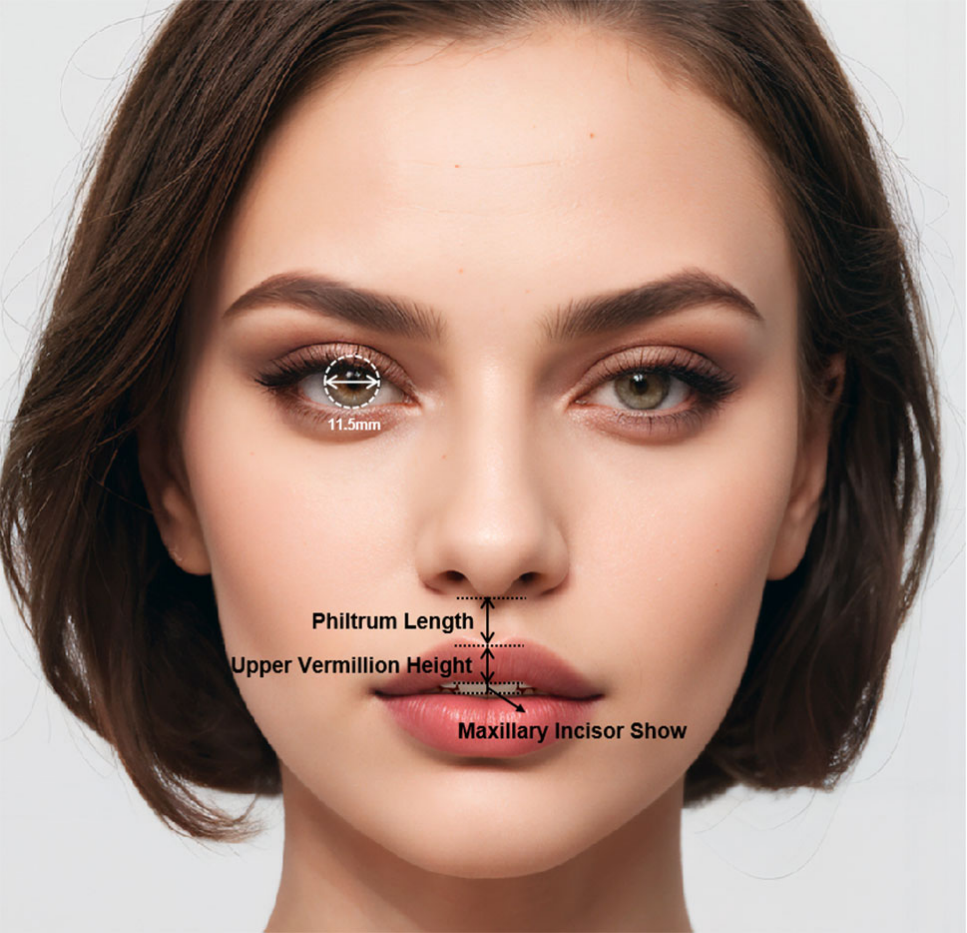
Different variables altered to create different philtrum lengths

**Fig. 2:**
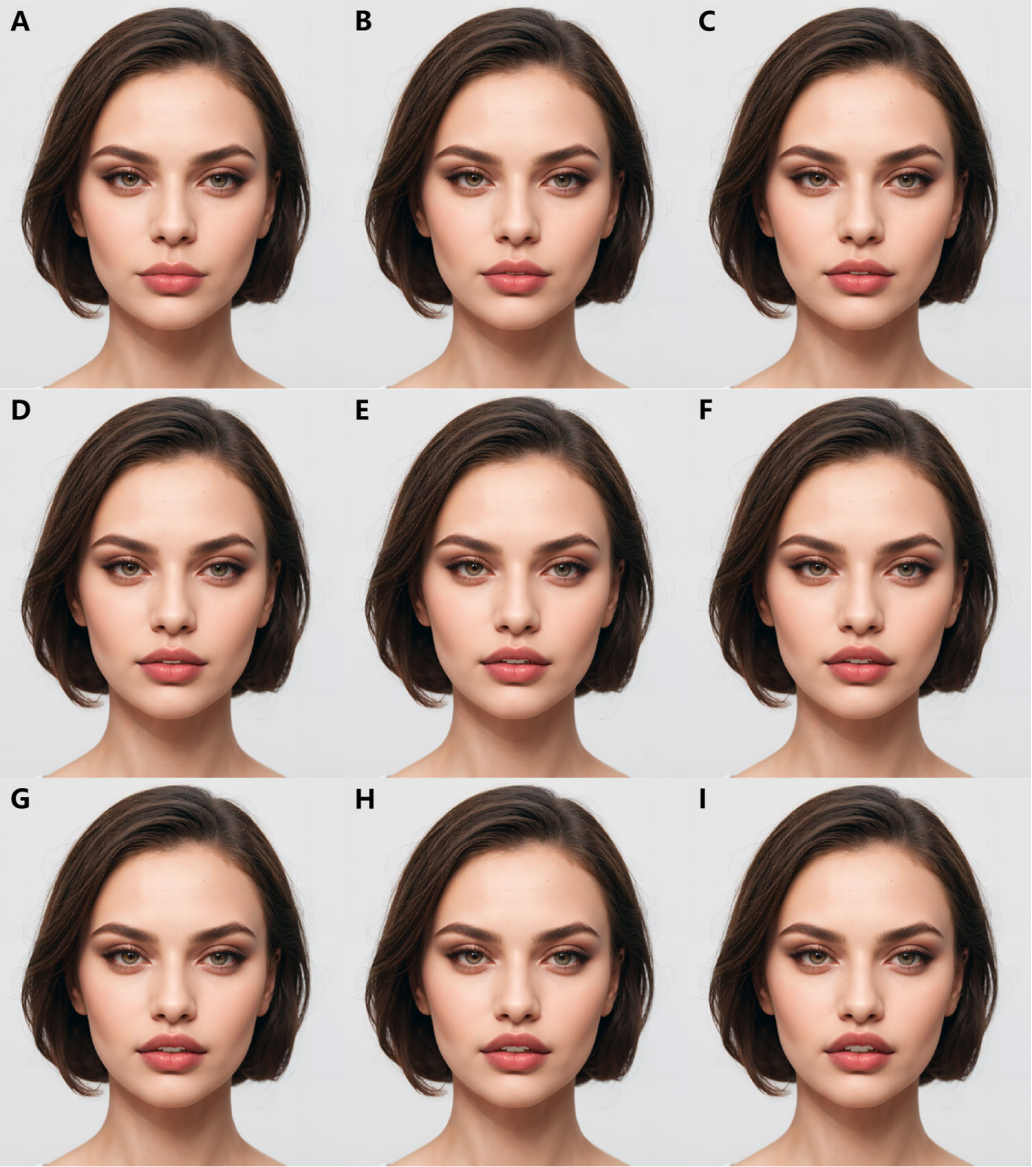
Varying levels of maxillary incisor show

Following this, the images were carefully edited using Adobe Photoshop (Adobe Systems Inc, San Jose, CA, USA) to simulate the effects of varying degrees of a bullhorn lip lift. This involved adjustments to the philtrum length, upper vermillion height, and the amount of maxillary incisor show. Specifically, for every 0.65 mm decrease in philtrum length, there were a corresponding increase of 0.15 mm in upper vermillion height and an increase of Level 2 in maxillary incisor show. Through this process, a total of nine images were produced for each ethnic group, representing nine different maxillary incisor shows from none (= Level 1) to excessive (= Level 9) (Figs. 1 and 2).

### Online Survey

The study's subsequent phase involved gathering participant feedback via an online survey. This survey was developed and distributed using Google Forms (Google, Mountain View, CA, USA) and Wen Juan Xing (Changsha Ranxing Information Technology Co., Ltd., Changsha, China). The survey distribution targeted a diverse audience, including both medical professionals and laypersons, who were invited through direct email invitations, social media platforms, and professional networks. Follow-up reminders were sent twice to improve response rates.

The survey consisted of demographic questions collecting information about the participants' gender, age, country of origin, ethnic background, and professional affiliation (medical professional or layperson). Medical professionals were further asked to indicate their specialty. Participants were instructed to evaluate the aesthetic appeal of lips in each image using a 5-point Likert scale (1: "very unattractive" to 5: "very attractive").

The survey presented a randomized set of 45 images (nine images per ethnic group) and allowed participants to progress at their own pace. Participants were also permitted to review and revise their responses during the survey. A response rate cannot be calculated, as the survey also used social media platforms and professional networks.

### Statistical Analysis

To analyze the collected data, statistical analysis was performed using SPSS version 27.0 (IBM Corp., Armonk, NY, USA). Intrarater reliability was assessed by inviting 62 randomly selected participants to retake the survey after a 4-week washout period. The intraclass correlation coefficient (ICC) was calculated to determine the reliability of the participants' ratings. To assess the statistical significance of differences in the attractiveness scores of lips across varying levels of maxillary incisor show, the Kruskal–Wallis nonparametric test was employed. Results were considered statistically significant at a probability level of ≤ 0.05, which was used to guide the study's conclusions.

## Results

### Respondent Characteristics

A total of 570 respondents participated in this study. Table 1 summarizes the demographic characteristics of the respondents. In terms of ethnic background, two participants (0.3%) were categorized as “Other” (one Native American and one Maori). Due to the insufficient sample size in this category, meaningful statistical analysis could not be performed. Therefore, data from the group, along with those who selected “Prefer not to say” (5 participants, 0.8% by gender, and 4 participants, 0.7% by ethnicity), were excluded from the statistical analysis of lip attractiveness rating scores across different levels of maxillary incisor show within each gender or ethnic group. When classified by occupation, 72 respondents (12.6%) were medical professionals, while 498 (87.3%) were laypersons. Among the medical professionals, there were 3 plastic surgeons, 1 dermatologist, and 3 dental specialists, with the remaining individuals being other clinical or allied health professionals.

**Table 1:** Respondent demographic profile (n = 570)

Demographic variables	N
*Gender*	
Male	246 (43.1%)
Female	318 (55.7%)
Divers	1 (0.1%)
Other	0 (0%)
Prefer not to say	5 (0.8%)
*Age range (years)*	
<20	15 (2.6%)
20–29	126 (22.1%)
30–39	140 (24.5%)
40–49	60 (10.5%)
50–59	78 (13.6%)
60–69	95 (16.6%)
≥70	56 (9.8%)
*Ethnic background*	
African	53 (9.2%)
Asian	224 (39.2%)
Caucasian	171 (30.0%)
Hispanic/Latino	42 (7.3%)
Middle eastern	45 (7.8%)
Mixed or multiple ethnicities	29 (5.0%)
Other	2 (0.3%)
Prefer not to say	4 (0.7%)
*Profession*	
Medical professional	72 (12.6%)
Layperson	498 (87.3%)

### Intrarater Reliability Assessment

The intrarater reliability of participants' ratings was good, with an ICC of 0.753 (95% confidence interval: 0.734–0.771). The responses from the reliability test were excluded from the main study.

### Aesthetic Appeal Rating of Lips

**Fig. 3:**
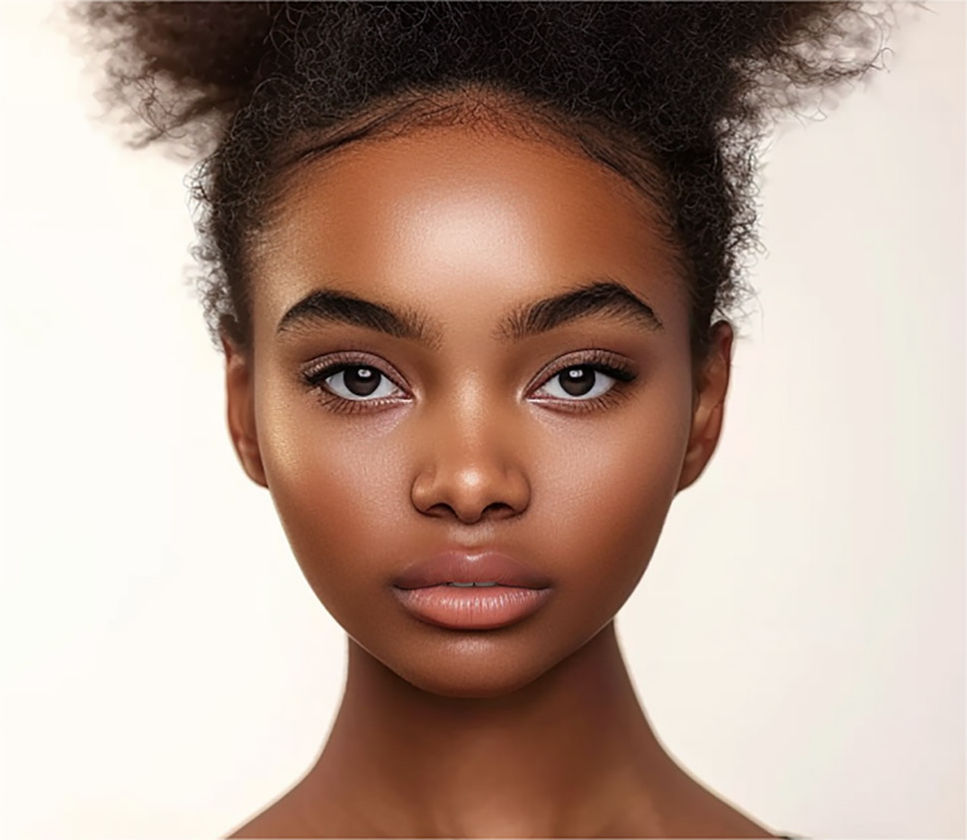
The most aesthetically preferred lip position for the African image, as determined by the overall population of raters, is shown with a level 3 incisor show. This lip position received a mean attractiveness score of 3.13 ± 1.04, indicating it was the most favored among the presented options for this ethnic group

**Fig. 4:**
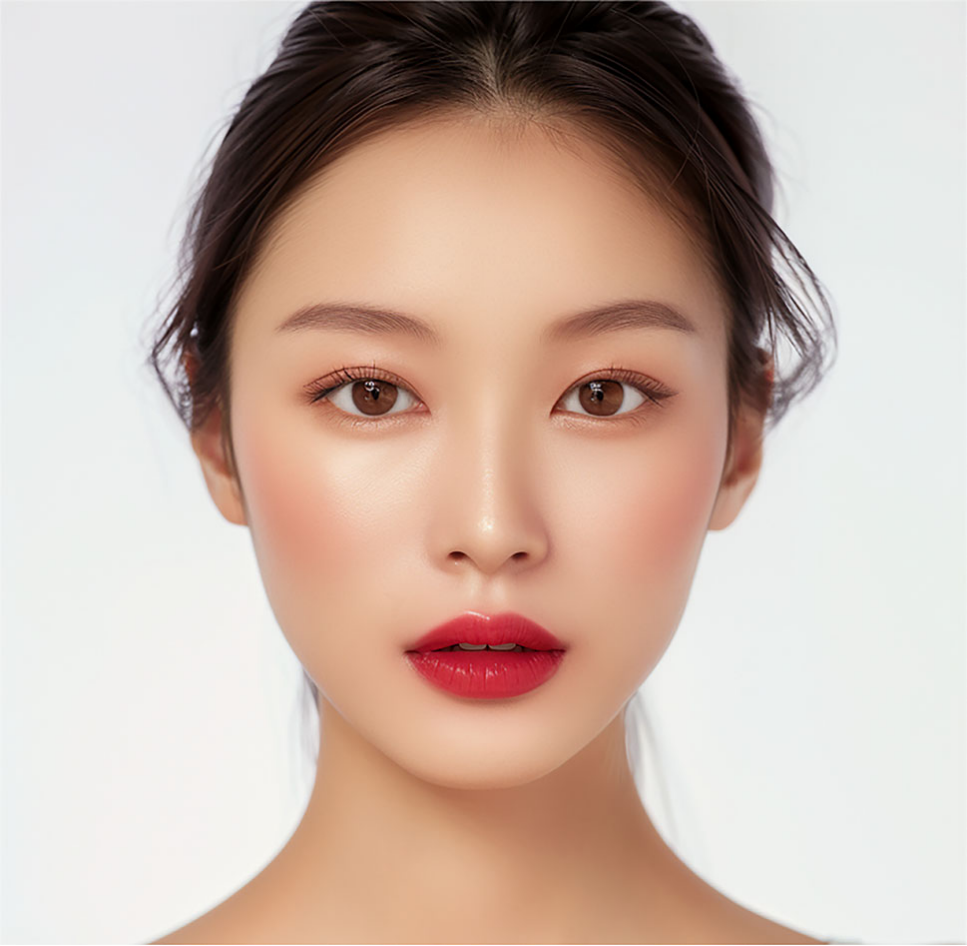
The most aesthetically preferred lip position for the Asian image is illustrated with a level 3 incisor show. This lip configuration achieved the highest mean score of 3.64 ± 0.87, reflecting its preference among the overall population of raters for this ethnic group

**Fig. 5:**
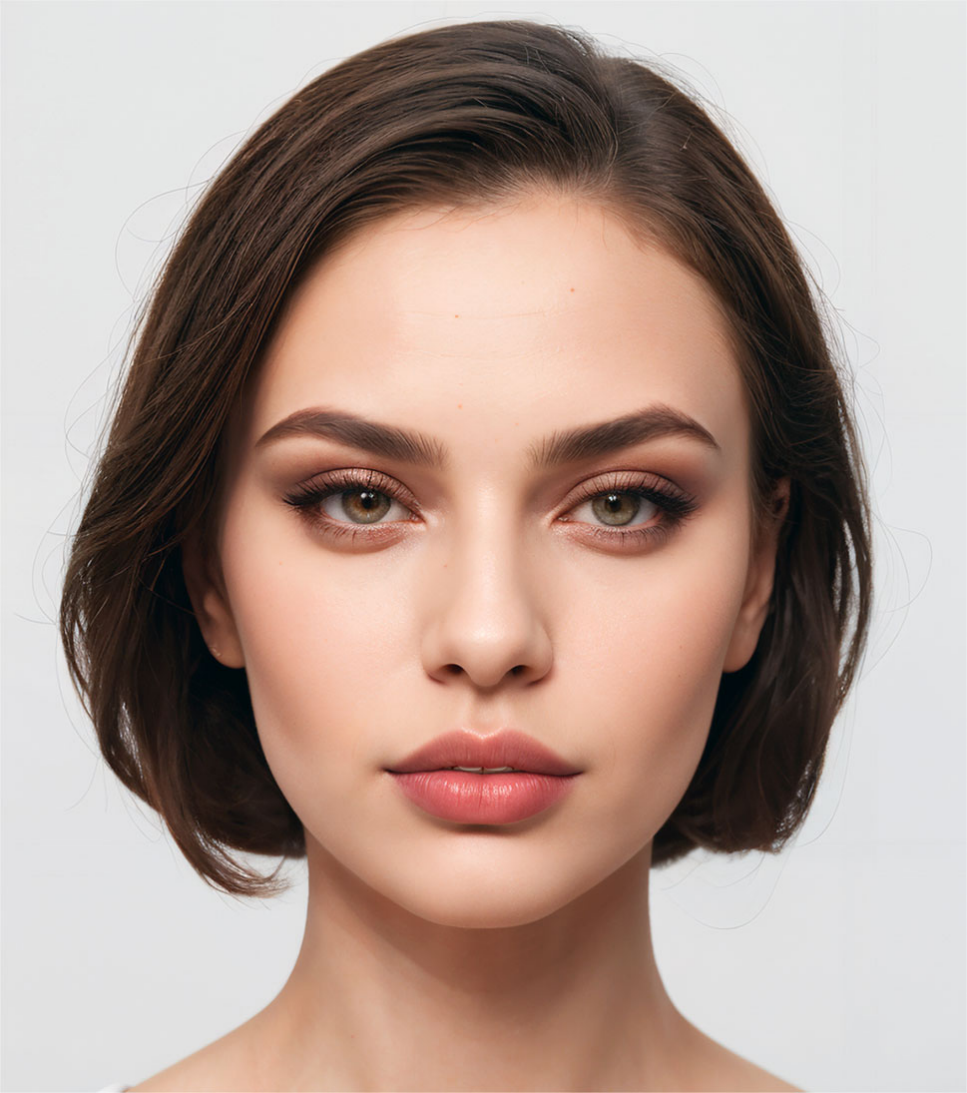
The Caucasian image with a level 2 incisor show represents the most aesthetically preferred lip position, receiving the highest mean score of 4.04 ± 0.79 among the overall population of raters for this group

**Fig. 6:**
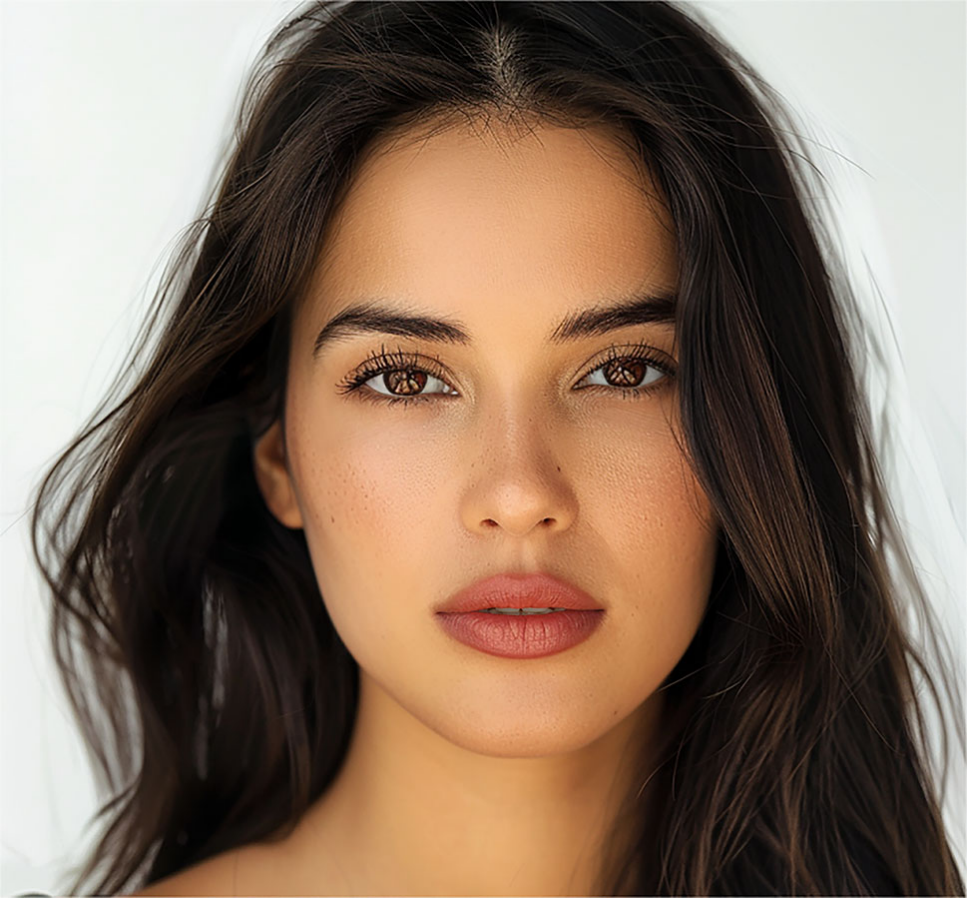
Latino image displays the most favored lip position, featuring a level 2 incisor show. This lip position was rated highest by the overall population of raters, with a mean score of 3.92 ± 0.84

**Fig. 7:**
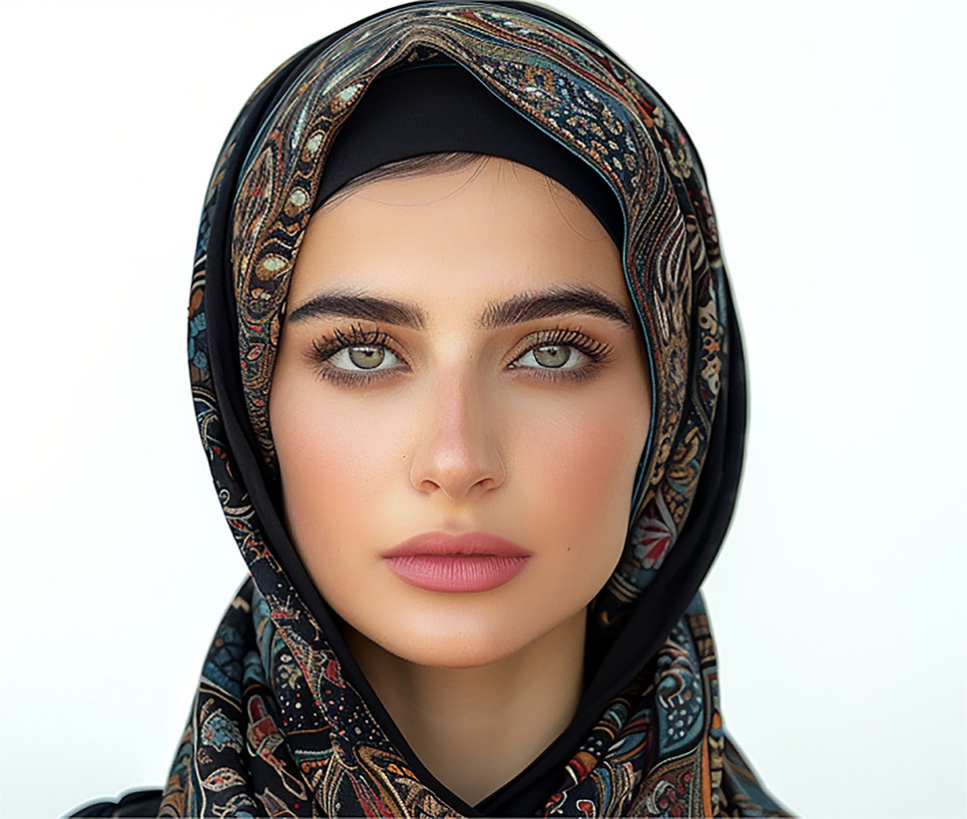
The most aesthetically preferred lip position for the Middle Eastern image is depicted with a level 1 incisor show. This position received a mean score of 3.49 ± 0.90, making it the most favored among the presented options for this ethnic group

When considering the overall population of raters, without stratification by gender, ethnicity, or age, the most preferred lips in African and Asian images were those of the Level 3 incisor show, with mean scores of 3.13 ± 1.04 and 3.64 ± 0.87, respectively. In Caucasian and Latino images, the most favored lips were those of the Level 2 incisor show, with mean scores of 4.04 ± 0.79 and 3.92 ± 0.84, respectively. For Middle Eastern images, the highest rated lips were in the Level 1 incisor show image (mean score 3.49 ± 0.90) (Figs. 3, 4, 5, 6 and 7). The least favored lips, except in the African images where the Level 8 incisor show image was rated lowest (mean score 2.83 ± 1.08), were consistently those of the Level 9 incisor show, with the Level 8 incisor show lips being the second least favored. In each ethnicity of images, the differences in aesthetic preferences for lip attractiveness across varying levels of maxillary incisor show were statistically significant (p < 0.001) (Table 2). When analyzing by gender, the most and least preferred lips remained consistent, except for male respondents who most favored the Level 2 incisor show in the African images (Table 3).

**Table 2:** Mean lip aesthetic rating scores for the respective incisor shows

Image ethnicity	Level 1	Level 2	Level 3	Level 4	Level 5	Level 6	Level 7	Level 8	Level 9	*P*
African	3.05±1.06	3.12±1.06	3.13±1.04	3.04±1.05	3.09±1.10	3.03±1.01	2.98±1.05	2.83±1.08	2.99±1.14	<0.001*
Asian	3.57±0.91	3.53±0.93	3.64±0.87	3.42±0.92	3.50±0.94	3.29±0.95	3.18±1.01	3.00±1.08	2.78±1.11	0.000*
Caucasian	3.92±0.85	4.04±0.79	3.93±0.85	3.77±0.87	3.74±1.02	3.75±1.02	3.48±1.09	3.31±1.15	3.04±1.21	0.000*
Latino	3.90±0.86	3.92±0.84	3.88±0.86	3.84±0.85	3.81±0.88	3.79±0.89	3.70±0.97	3.49±1.02	3.26±1.10	0.000*
Middle Eastern	3.49±0.90	3.37±0.93	3.36±0.95	3.32±0.92	3.29±0.96	3.21±0.95	3.34±0.98	3.07±1.05	2.89±1.06	0.000*

The values are listed separately by the ethnicity of images. Values are presented as mean±standard deviation. The *P* value indicates the significance of the difference among the incisor shows. *P<0.05

**Table 3:** Mean lip aesthetic rating scores for the respective incisor shows

Image ethnicity	Participant gender	Level 1	Level 2	Level 3	Level 4	Level 5	Level 6	Level 7	Level 8	Level 9	*P*
African	Male	2.98±1.02	3.08±0.99	3.05±1.00	3.00±0.99	2.99±1.01	2.91±0.95	2.87±1.02	2.72±1.06	2.90±1.10	0.002*
	Female	3.10±1.10	3.14±1.11	3.19±1.07	3.07±1.10	3.15±1.16	3.12±1.05	3.05±1.08	2.91±1.09	3.07±1.18	0.051
Asian	Male	3.54±0.96	3.53±0.96	3.59±0.86	3.35±0.96	3.46±0.94	3.20±1.00	3.07±1.07	2.93±1.14	2.69±1.15	0.000*
	Female	3.59±0.88	3.53±0.90	3.69±0.88	3.46±0.89	3.54±0.94	3.36±0.91	3.26±0.95	3.05±1.03	2.85±1.06	0.000*
Caucasian	Male	3.98±0.83	4.04±0.78	3.93±0.83	3.73±0.90	3.68±1.04	3.69±1.06	3.43±1.13	3.22±1.19	2.94±1.28	0.000*
	Female	3.88±0.86	4.04±0.79	3.94±0.88	3.81±0.85	3.79±1.00	3.81±0.98	3.53±1.06	3.38±1.11	3.11±1.16	0.000*
Latino	Male	3.94±0.83	3.95±0.86	3.90±0.89	3.93±0.80	3.87±0.88	3.89±0.86	3.77±1.00	3.54±1.06	3.33±1.16	<0.001*
	Female	3.87±0.89	3.91±0.83	3.86±0.84	3.79±0.88	3.78±0.88	3.71±0.90	3.65±0.95	3.45±0.99	3.20±1.05	0.000*
Middle Eastern	Male	3.49±0.93	3.33±0.97	3.32±1.01	3.28±0.95	3.24±1.01	3.21±0.97	3.33±0.97	3.04±1.11	2.83±1.15	<0.001*
	Female	3.49±0.88	3.41±0.90	3.39±0.90	3.36±0.89	3.33±0.92	3.21±0.93	3.37±0.99	3.09±0.99	2.93±0.98	<0.001*

The values are listed separately by the ethnicity of images and the gender of participants. Values are presented as mean±standard deviation. The *P* value indicates the significance of the difference among the incisor shows. *P<0.05

### Influence of Age

In the various ethnicity of images, the most preferred lips in different age groups were still concentrated in the Level 1, Level 2, and Level 3 incisor show images, with the exception of the 50–59 age group, which favored the Level 4 incisor show in Latino images (mean score 4.09±0.74). In African images, although the differences in scores across various incisor shows in each age group were not statistically significant (p > 0.05), it was consistently observed that the lowest scores in each age group were found in the Level 8 incisor show lips. In the other ethnic images, the least favored lips of each age range were still primarily found in the Level 9 incisor show, with the Level 8 incisor show being predominantly the second least favored (Table 4).

**Table 4:** Mean lip aesthetic rating scores for the respective incisor shows

Image ethnicity	Participant age range (years)	Level 1	Level 2	Level 3	Level 4	Level 5	Level 6	Level 7	Level 8	Level 9	*P*
African	<20	2.80±0.94	3.20±1.01	3.27±1.10	3.07±0.96	2.87±1.19	2.87±0.99	2.73±1.16	2.13±0.83	3.07±1.22	0.142
	20–29	2.98±1.14	3.13±1.16	3.10±1.07	2.98±1.11	3.02±1.11	2.94±1.05	2.87±1.13	2.73±1.12	2.93±1.21	0.148
	30–39	2.94±1.10	3.04±1.08	3.00±1.06	2.98±1.09	3.01±1.14	2.88±1.03	2.84±1.04	2.65±1.10	2.84±1.19	0.068
	40–49	3.22±0.90	3.17±0.96	3.18±0.91	3.13±1.00	3.18±1.05	3.10±0.90	3.10±1.00	2.90±1.04	3.05±1.10	0.701
	50–59	3.23±0.98	3.32±0.95	3.31±0.97	3.22±0.95	3.28±1.01	3.24±0.93	3.24±0.91	3.10±0.98	3.14±1.03	0.921
	60–69	3.05±1.07	3.05±1.08	3.14±1.11	3.04±1.03	3.09±1.09	3.13±1.05	3.03±1.11	2.98±1.06	3.09±1.10	0.992
	≥70	3.09±1.08	3.04±1.01	3.20±1.03	3.02±1.05	3.07±1.13	3.09±1.03	3.02±0.98	2.95±1.05	3.04±1.13	0.961
Asian	<20	2.80±0.94	2.93±1.03	3.27±1.10	2.73±1.10	2.87±1.19	2.87±1.13	2.60±1.18	2.07±1.22	1.80±0.86	0.009*
	20–29	3.53±0.95	3.55±0.94	3.58±0.92	3.37±0.91	3.44±1.00	3.06±0.94	2.91±1.09	2.68±1.11	2.45±1.11	0.000*
	30–39	3.59±0.87	3.46±0.92	3.74±0.82	3.44±0.86	3.50±0.95	3.26±0.93	3.06±0.92	2.86±1.02	2.61±1.07	0.000*
	40–49	3.53±0.81	3.52±0.87	3.63±0.76	3.35±0.88	3.53±0.75	3.23±0.91	3.12±0.92	3.07±1.10	2.70±0.96	<0.001*
	50–59	3.56±0.88	3.62±0.87	3.65±0.85	3.49±1.00	3.58±0.95	3.44±0.95	3.44±1.03	3.28±1.01	3.14±1.08	0.026*
	60–69	3.55±0.92	3.57±0.95	3.61±0.89	3.47±0.90	3.55±0.87	3.46±0.90	3.46±0.90	3.28±1.00	3.13±1.00	0.016*
	≥70	3.88±0.97	3.70±0.95	3.70±0.97	3.52±0.93	3.59±1.01	3.52±1.01	3.48±0.95	3.39±0.95	3.20±1.14	0.020*
Caucasian	<20	4.13±0.64	3.67±0.82	3.53±0.92	3.27±0.88	3.20±1.52	3.27±1.28	2.40±1.40	2.33±1.29	2.13±1.46	<0.001*
	20–29	3.94±0.86	4.04±0.77	3.98±0.85	3.74±0.85	3.63±1.00	3.71±1.01	3.24±1.11	3.05±1.13	2.75±1.21	0.000*
	30–39	4.01±0.82	4.04±0.75	3.96±0.86	3.76±0.86	3.78±1.00	3.82±1.02	3.44±1.05	3.16±1.17	2.79±1.24	0.000*
	40–49	3.92±0.91	4.20±0.76	4.07±0.80	3.85±0.92	3.90±0.99	3.88±1.01	3.55±1.14	3.32±1.05	3.17±1.15	<0.001*
	50–59	3.94±0.89	4.08±0.82	3.85±0.87	3.85±0.85	3.77±1.01	3.77±0.95	3.67±1.04	3.64±1.09	3.28±1.15	<0.001*
	60–69	3.72±0.83	3.95±0.79	3.79±0.87	3.72±0.91	3.67±1.06	3.63±1.08	3.64±0.99	3.47±1.10	3.37±1.11	0.021*
	≥70	3.91±0.84	4.05±0.84	4.09±0.79	3.89±0.85	3.96±0.87	3.86±0.90	3.86±0.94	3.80±0.98	3.52±1.01	0.140
Latino	<20	4.40±0.63	3.87±0.83	3.60±0.91	3.93±0.59	3.80±0.78	3.80±0.78	3.80±0.68	3.07±1.10	2.40±0.99	<0.001*
	20–29	3.91±0.83	3.93±0.92	3.93±0.83	3.84±0.85	3.82±0.90	3.76±0.86	3.66±0.92	3.41±1.01	3.13±1.10	<0.001*
	30–39	3.96±0.83	3.91±0.83	3.85±0.84	3.83±0.81	3.79±0.90	3.80±0.89	3.65±1.02	3.39±1.06	3.06±1.15	<0.001*
	40–49	3.82±0.95	4.02±0.87	3.82±0.85	3.77±0.85	3.83±0.85	3.77±0.87	3.83±0.92	3.53±1.02	3.33±1.05	0.022*
	50–59	3.99±0.76	4.01±0.73	4.05±0.75	4.09±0.74	3.88±0.79	3.95±0.75	3.77±0.94	3.71±0.91	3.50±0.96	<0.001*
	60–69	3.73±0.96	3.80±0.87	3.76±0.96	3.72±0.96	3.66±0.97	3.73±0.97	3.63±1.05	3.53±1.04	3.39±1.06	0.166
	≥70	3.84±0.89	3.95±0.77	3.91±0.92	3.82±0.88	4.00±0.79	3.75±1.00	3.80±1.00	3.66±1.00	3.61±1.06	0.529
Middle Eastern	<20	3.60±0.63	3.00±0.66	2.93±0.96	3.20±0.78	2.87±1.06	2.53±0.92	2.80±1.01	2.13±0.99	2.20±1.27	0.001*
	20–29	3.30±0.97	3.20±0.98	3.17±0.94	3.14±0.87	3.13±0.98	3.02±0.89	3.11±1.02	2.77±1.00	2.43±0.98	<0.001*
	30–39	3.39±0.88	3.23±0.92	3.21±0.93	3.11±0.91	3.11±0.88	3.02±0.94	3.26±0.97	2.86±1.02	2.66±0.96	<0.001*
	40–49	3.53±0.85	3.48±0.91	3.33±0.86	3.22±0.87	3.42±0.98	3.23±0.95	3.42±0.93	3.17±0.99	2.93±0.99	0.012*
	50–59	3.73±0.83	3.58±0.80	3.62±0.89	3.58±0.93	3.56±0.86	3.50±0.85	3.53±0.88	3.40±1.02	3.22±0.99	0.077
	60–69	3.60±0.93	3.53±0.94	3.54±1.01	3.56±0.90	3.44±1.00	3.44±0.98	3.53±0.97	3.32±0.99	3.35±0.99	0.482
	≥70	3.57±0.91	3.57±0.97	3.63±0.96	3.61±0.97	3.39±1.02	3.48±0.93	3.57±0.97	3.55±0.95	3.39±1.04	0.854

The values are listed separately by the ethnicity of images and the age range of participants. Values are presented as mean±standard deviation. The *P* value indicates the significance of the difference among the incisor shows. *P<0.05

### Influence of Respondents‘ Ethnic Background

When the analysis was further stratified by respondents' ethnic background, we observed that in African images, only the Caucasian group exhibited statistically significant differences in scores across varying incisor show levels (p < 0.001). In contrast, there were no statistically significant differences in scores across different incisor show levels for other respondents' ethnic groups (p > 0.05). However, a consistent trend was noted, with the lowest scores being associated with the Level 8 incisor show lips. In Asian, Caucasian, Latino, and Middle Eastern images, the most favorable lips for various ethnic respondents were still concentrated in the Level 1, Level 2, and Level 3 incisor shows, except in the Caucasian images where African respondents gave the highest score to the Level 4 incisor show lips (mean score 4.04±0.85). The least favored lips remained in the Level 9 incisor show, with the Level 8 incisor show being predominantly the second least favored (Table 5).

**Table 5:** Mean lip aesthetic rating scores for the respective incisor shows

Image ethnicity	Participant ethnicity	Level 1	Level 2	Level 3	Level 4	Level 5	Level 6	Level 7	Level 8	Level 9	*P*
African	African	3.55±1.10	3.51±1.03	3.51±0.99	3.58±0.97	3.68±1.03	3.51±0.99	3.64±0.98	3.42±1.08	3.51±1.19	0.943
	Asian	2.60±1.02	2.65±1.02	2.72±1.04	2.61±1.01	2.64±1.07	2.68±1.00	2.60±0.98	2.51±1.00	2.53±1.08	0.356
	Caucasian	3.29±0.97	3.35±0.92	3.32±0.96	3.22±0.94	3.29±1.01	3.16±0.95	3.09±0.96	2.89±0.99	3.25±1.03	<0.001*
	Hispanic/Latino	3.55±0.80	3.64±0.96	3.60±0.86	3.48±0.92	3.64±0.82	3.38±0.83	3.36±0.98	3.19±1.11	3.38±1.01	0.540
	Middle Eastern	2.98±0.99	3.29±1.01	3.27±0.96	3.09±1.04	2.93±1.07	2.98±0.99	2.98±1.20	2.73±1.12	2.87±1.14	0.143
	Mixed or multiple ethnicities	3.52±0.99	3.66±0.97	3.62±0.94	3.62±1.02	3.62±0.90	3.55±0.91	3.41±1.02	3.41±1.12	3.72±1.13	0.951
Asian	African	3.66±0.94	3.68±0.94	3.74±0.74	3.57±0.97	3.60±0.88	3.30±0.89	3.30±1.01	3.13±1.08	3.11±1.12	0.002*
	Asian	3.43±0.98	3.42±0.99	3.43±0.93	3.37±0.91	3.42±0.98	3.32±0.96	3.26±0.97	3.10±1.01	2.84±1.08	<0.001*
	Caucasian	3.74±0.79	3.65±0.87	3.89±0.73	3.43±0.90	3.60±0.88	3.26±0.94	3.14±0.97	2.87±1.11	2.68±1.05	0.000*
	Hispanic/Latino	3.76±0.91	3.76±0.91	3.71±1.04	3.64±0.85	3.48±1.02	3.36±0.98	3.17±1.10	3.12±1.11	2.81±1.15	<0.001*
	Middle Eastern	3.40±0.94	3.29±0.82	3.56±0.89	3.13±1.10	3.29±1.04	3.07±1.14	2.71±1.18	2.73±1.21	2.44±1.32	<0.001*
	Mixed or multiple ethnicities	3.52±0.79	3.59±0.73	3.83±0.71	3.55±0.69	3.69±0.76	3.45±0.78	3.38±0.90	3.10±1.15	2.86±1.09	0.007*
Caucasian	African	3.94±0.86	4.02±0.75	4.00±0.88	4.04±0.85	3.85±0.95	3.98±0.91	3.79±0.99	3.51±1.14	3.47±1.17	0.047*
	Asian	3.86±0.87	3.97±0.84	3.94±0.85	3.77±0.91	3.86±0.94	3.85±0.94	3.65±1.02	3.56±1.05	3.27±1.14	<0.001*
	Caucasian	3.90±0.83	4.04±0.71	3.84±0.84	3.65±0.81	3.56±1.08	3.51±1.09	3.23±1.05	2.98±1.14	2.70±1.11	0.000*
	Hispanic/Latino	4.14±0.84	4.36±0.79	4.10±0.96	3.90±0.85	3.81±1.22	3.90±1.14	3.52±1.23	3.17±1.27	2.98±1.42	<0.001*
	Middle Eastern	4.04±0.88	4.02±0.75	3.91±0.90	3.69±0.97	3.53±1.14	3.71±1.12	3.20±1.34	3.04±1.26	2.71±1.42	<0.001*
	Mixed or multiple ethnicities	4.00±0.80	4.17±0.71	4.17±0.60	3.93±0.70	4.07±0.65	4.00±0.66	3.48±1.09	3.59±1.05	3.10±1.21	<0.001*
Latino	African	3.94±0.84	3.87±0.98	3.92±0.78	3.91±0.86	3.81±1.00	3.75±0.76	3.91±0.93	3.70±0.93	3.62±1.00	0.657
	Asian	3.73±0.85	3.78±0.83	3.71±0.87	3.65±0.85	3.68±0.82	3.63±0.89	3.54±0.99	3.40±0.99	3.22±1.01	<0.001*
	Caucasian	3.94±0.89	3.95±0.83	3.89±0.86	3.87±0.85	3.81±0.94	3.82±0.92	3.66±0.97	3.39±1.07	3.09±1.14	0.000*
	Hispanic/Latino	4.07±0.84	4.10±0.79	4.17±0.76	4.17±0.73	4.07±0.84	4.14±0.81	4.02±0.81	3.88±0.89	3.52±1.15	0.123
	Middle Eastern	3.98±0.81	4.16±0.77	4.07±0.89	4.02±0.87	4.00±0.83	3.91±0.95	3.82±1.05	3.36±1.17	3.09±1.24	<0.001*
	Mixed or multiple ethnicities	4.31±0.71	4.28±0.70	4.07±0.75	4.21±0.62	4.07±0.65	4.03±0.63	3.97±0.82	3.90±0.86	3.52±1.02	0.031*
Middle Eastern	African	3.43±0.95	3.40±0.97	3.34±0.94	3.47±0.91	3.40±0.95	3.45±0.93	3.32±1.07	3.17±1.05	3.08±1.12	0.521
	Asian	3.29±0.87	3.23±0.91	3.25±0.93	3.19±0.93	3.19±0.91	3.08±0.91	3.25±0.92	3.06±0.96	2.95±0.96	<0.001*
	Caucasian	3.66±0.89	3.45±0.87	3.46±0.91	3.43±0.89	3.35±0.92	3.25±0.95	3.46±0.92	3.04±1.07	2.83±1.07	<0.001*
	Hispanic/Latino	3.69±0.92	3.74±1.04	3.52±1.11	3.43±0.99	3.50±1.09	3.31±1.00	3.38±1.13	3.36±1.12	2.79±1.20	0.010*
	Middle Eastern	3.56±0.89	3.36±1.00	3.18±0.98	3.13±0.92	3.11±1.19	3.11±1.09	3.20±1.22	2.67±1.17	2.58±1.23	<0.001*
	Mixed or multiple ethnicities	3.66±0.94	3.41±1.02	3.59±1.02	3.41±0.91	3.41±0.98	3.45±0.91	3.48±0.99	3.34±1.11	3.00±1.13	0.456

The values are listed separately by the ethnicity of images and the ethnicity of participants. Values are presented as mean±standard deviation. The *P* value indicates the significance of the difference among the incisor shows. *P<0.05

## Discussion

This study was conducted to explore the effect of philtrum length on the overall facial aesthetic appeal. With the increasing popularity of lip augmentation procedures, particularly the bullhorn lip lift, there is a growing need to understand the ideal proportions that contribute to facial attractiveness across different ethnic groups. Utilizing artificial intelligence to generate and modify facial images, the study simulated various degrees of philtrum shortening and corresponding changes in the upper vermillion height and maxillary incisor show. These images were then assessed by a diverse group of participants to evaluate the aesthetic appeal of different configurations.

The findings revealed that a moderate shortening of the philtrum, which results in a balanced display of teeth, is generally preferred. Specifically, among Caucasian images, an incisor show of approximately Level 2 was found to be most attractive. This suggests that while some degree of tooth display enhances the perception of youthfulness and vitality, excessive display can detract from overall facial harmony. The study highlights that an overly elongated philtrum is often considered unattractive.

The results of this study have important implications for surgeons. The bullhorn lip lift, a procedure designed to reduce the length of the philtrum and enhance lip prominence, must be approached with a nuanced understanding of the patient's ethnic background and personal preferences. The study’s findings suggest that achieving a balance between philtrum length and maxillary incisor show is critical to enhancing facial attractiveness, but this balance varies depending on the cultural and ethnic context of the patient. For Caucasian patients, a slight increase in tooth display—around Level 2—was considered attractive. This subtle adjustment can enhance the youthful appearance of the patient without making the teeth overly prominent, which could lead to an exaggerated or unnatural look. Similarly, among Asian and Middle Eastern participants, a balanced amount of tooth display was also favored, though the specific millimeter preference varied slightly, reflecting the nuanced differences in aesthetic ideals across these groups. For Latino and African participants, a slightly more prominent display of teeth was generally well received, yet excessive exposure still led to lower attractiveness ratings, underscoring the universal preference for moderation. The concept of "less is more" remains particularly relevant across these ethnicities; while a slight reduction in philtrum length can rejuvenate the appearance and enhance facial harmony, excessive reduction risks overexposing the teeth, which is often perceived negatively regardless of ethnic background [20, 21]. This finding highlights the importance of tailoring aesthetic procedures like the bullhorn lip lift to the specific cultural and individual preferences of patients to achieve the most natural and appealing results. Surgeons should also consider the aging implications of philtrum length. A longer philtrum is often associated with aging due to the natural elongation that occurs with time.[22] Therefore, patients seeking a more youthful appearance may benefit from a bullhorn lip lift. However, the extent of the procedure should be carefully planned. Over-correction can lead to an unnatural smile that might age poorly or seem incongruent with the rest of the facial features. A thorough consultation process that includes a detailed discussion of the patient’s goals and expectations is essential. By aligning surgical outcomes with the patient’s individual perception of beauty, surgeons can enhance patient satisfaction and achieve more natural, harmonious results. The growing diversity in patient populations also presents both challenges and opportunities for aesthetic medicine professionals.[23] Understanding the varied needs and preferences of patients from different ethnic and cultural backgrounds is crucial for delivering personalized and culturally sensitive treatments. This study provides valuable insights into how these differences manifest in the context of lip aesthetics, and it highlights the need for a more nuanced approach to facial rejuvenation procedures.

Teeth, as a visible feature in the smile, play a crucial role in this perception. The study’s findings align with existing literature that underscores the importance of teeth.[24] In humans, teeth serve not only functional and health-related purposes but also act as significant indicators of attractiveness. Previous literature states that the presence of a balanced gingival display, particularly during dynamic smiling, is often perceived as attractive, suggesting that the interplay between teeth visibility and lip position is critical in determining the overall aesthetic of the smile.[21] Interestingly, an excessive display of teeth was consistently rated as less attractive. This may be due to the association of overly visible teeth with certain negative connotations, such as aggressiveness or poor health, which can disrupt the harmony of the face.

In a broader context, the findings of this investigation contribute to our understanding of how beauty standards are constructed and perceived. The perception of beauty is a dynamic process, influenced by cultural norms, social interactions and individual experiences. As the patient population in aesthetic medicine becomes increasingly diverse, it is essential for practitioners to recognize and respect these differences. A one-size-fits-all approach is no longer sufficient; instead, a personalized approach that takes into account the unique cultural and ethnic background of each patient is necessary to achieve the best outcomes.

Furthermore, the psychosocial impact of aesthetic procedures cannot be ignored. The way individuals perceive their own beauty, particularly in the context of their smile, can have profound effects on their self-esteem and social interactions. A recent study by Van der Geld et al. on smile attractiveness highlights the importance of teeth and lip position in self-perception.[20] Their findings suggest that individuals who perceive their smiles as attractive tend to have higher self-esteem and more positive social interactions. This underscores the importance of achieving a balanced and natural-looking smile through aesthetic procedures, as the benefits extend beyond physical appearance to encompass psychological well-being and social confidence.

This study's key strengths include its large sample size, which enhances the reliability and generalizability of the findings. The diverse participant pool, including both medical professionals and laypersons, provides a broad perspective on aesthetic preferences. Another major strength is the study's ethnically diverse approach, which acknowledges varying beauty standards across cultures, offering insights applicable to a global population. The use of advanced AI technology (Midjourney) and image editing tools (Adobe Photoshop) allowed for precise control over variables such as philtrum length and incisor show, ensuring accurate visual stimuli and a controlled examination of aesthetic elements. The study’s findings are relevant across multiple disciplines, including plastic surgery, dermatology, orthodontics, and psychology, fostering interdisciplinary collaboration.

However, the reliance on static images to assess aesthetic appeal is a notable limitation, as it fails to capture the dynamic nature of facial expressions, particularly during smiling. Additionally, the use of AI-generated images, while allowing for controlled experimentation, may introduce biases by not fully capturing the nuances of real human faces. The study also does not deeply explore the cultural and social factors influencing participants' responses, which could oversimplify the complex nature of aesthetic preferences. The online survey format may introduce biases such as self-selection and might limit the depth of responses due to factors like screen quality or survey fatigue.

Furthermore, the analysis focused solely on lip length as a determinant of aesthetic appeal, without considering other factors such as lip volume, symmetry, and the overall harmony of facial proportions, which are also recognized contributors to perceived attractiveness. Future research should incorporate a more holistic approach by examining these additional parameters to better understand their combined influence on aesthetic evaluations.

In summary, while the study has significant strengths, including its large, diverse sample size and use of advanced technology, it also has limitations that suggest areas for further research, particularly in exploring dynamic facial expressions, cultural influences, psychological impacts, and a broader set of facial characteristics contributing to attractiveness.

## Conclusion

In conclusion, this study provides valuable insights into the complex relationship between philtrum length and the perception of beauty. The findings emphasize the importance of achieving a balanced aesthetic that aligns with the patient’s cultural background and personal preferences. For aesthetic professionals, these insights highlight the need for a tailored approach to facial rejuvenation, one that considers both the physical and psychological aspects of beauty. As our understanding of aesthetic perception continues to evolve, it is essential that these principles guide the practice of cosmetic surgery to ensure that the outcomes not only meet, but exceed, patient expectations.
